# Implementation, Acceptability, and Actions After Using an AI Workplace Health Kiosk in a Low-Resource Public School Workplace Setting: Cross-Sectional Pilot Study

**DOI:** 10.2196/87094

**Published:** 2026-04-01

**Authors:** Norvie Jalani, Jaybee Bazan, Jaime Kristoffer Punzalan

**Affiliations:** 1 Zamboanga City Medical Center, Santa Catalina, Zamboanga City, 7000, Philippines, 63 9175199629; 2 Ateneo De Zamboanga University School of Medicine, Zamboanga City, Philippines

**Keywords:** AI enabled, artificial intelligence enabled, Philippines, workplace, teachers, pilot

## Abstract

**Background:**

Artificial intelligence (AI)–enabled digital health kiosks are increasingly used in workplaces and communities to promote health awareness, especially in low- and middle-income countries. However, evidence on their real-world use, user acceptability, and immediate behavioral responses remains limited, especially outside formal clinical care.

**Objective:**

This study evaluated the implementation experience, user acceptability, and immediate self-reported actions associated with the use of an AI-enabled workplace health kiosk among public school teachers in an urban, low-resource setting in the Philippines.

**Methods:**

We conducted a study involving 384 teachers who used an AI health kiosk during wellness activities. The kiosk provided informational health indicators. Postuse surveys assessed usability; trust; privacy concerns; and self-reported actions, such as health consultations and sharing results. Analyses were descriptive and exploratory. The study did not evaluate diagnostic accuracy, clinical validity, disease prevalence, or health outcomes.

**Results:**

Most participants (162/189, 85.7%) rated the kiosk experience as good or excellent, and 93.1% (176/189) found it easy to use. Overall, trust in kiosk results was high, although 31.7% (60/189) of the participants expressed privacy concerns. After using the kiosk, 70.9% (134/189) of the participants consulted a health care professional, and 66.7% (126/189) made lifestyle changes. A small percentage (32/189, 16.9%) reported no follow-up actions, mainly due to uncertainty about the next steps. User feedback highlighted convenience and accessibility but also noted operational issues such as queuing and connectivity problems.

**Conclusions:**

In this workplace setting, an AI health kiosk was feasible, acceptable, and linked to immediate self-reported health actions. Findings are preliminary and context specific. Formal validation, follow-up studies, and further evaluation are needed before use in diagnostic, population health, or policy contexts.

## Introduction

Noncommunicable diseases (NCDs) continue to be the leading cause of illness and death worldwide, with a disproportionate impact in low- and middle-income countries (LMICs), where access to routine preventive screening is often limited. Workplace health promotion has been suggested as a practical approach to raise health awareness among employed adults, especially in the public sector where structured wellness programs are inconsistently implemented. Public school teachers in the Philippines constitute a large and stable workforce that is exposed to known occupational stressors and has limited access to employer-supported preventive health services. Previous studies have shown increased psychosocial strain and NCD risk factors among educators, highlighting the need for scalable, low-burden health awareness interventions within this group. Digital health kiosks have been increasingly used in nonclinical settings, such as workplaces and community spaces, to promote self-directed health engagement. Evidence from mostly high-income countries suggests that these kiosks can improve convenience, short-term awareness, and user participation; however, existing research has primarily focused on technical feasibility and measurement validation rather than real-world implementation and user acceptability, especially in LMICs. Recently, artificial intelligence (AI) has been integrated into health kiosk platforms to enable rule-based risk stratification, personalized feedback, and automated health messaging. These AI-enabled kiosks are designed as supportive, nondiagnostic tools that enhance health awareness without substituting clinical judgment. Across digital health initiatives, early deployment typically emphasizes implementation feasibility, usability, and trust before moving on to formal clinical validation and outcome assessment.

Concerns about data privacy, trust, and perceived surveillance have consistently been reported in workplace digital health initiatives, especially where boundaries between personal health information and employer oversight are unclear. These concerns are particularly prominent in public sector and institutional employment settings. In this context, this study examined the deployment of an AI-enabled health kiosk used during routine workplace wellness activities among public school teachers in an urban Philippine setting. This study did not assess diagnostic accuracy, disease prevalence, or clinical effectiveness. Instead, it addresses a gap in the literature by documenting real-world implementation, user acceptability, and immediate self-reported responses to an AI-enabled health kiosk in a nonclinical workplace setting within an LMIC context.

## Methods

### Design

This was a cross-sectional implementation and acceptability study conducted during scheduled workplace wellness activities in selected urban public schools in the Southern Philippines between July and October 2025. The study was designed to describe implementation, user acceptability, and immediate self-reported responses following interaction with an AI-enabled health kiosk in a real-world, nonclinical setting.

### Participants and Recruitment

A total of 384 public school teachers volunteered to participate, with recruitment coordinated by the Department of Education. Inclusion criteria included being an active public school teacher, aged 21 years or older, and willing to undergo screening and complete the postscreening survey. Teachers who were pregnant or acutely ill were excluded. Recruitment took place 6 months before the study began through school communications and announcements shared by school administrators during faculty meetings. These communications briefly explained the study’s goals, procedures, and the voluntary nature of participation. Participation was entirely voluntary, with no financial incentives, and all participants provided informed consent.

### AI-Enabled Health Kiosk

The DigiHealth system is a self-contained, AI-enabled health kiosk designed to support health awareness and engagement through automated health information delivery. The kiosk integrates basic physiological sensors commonly used in health kiosks, such as automated blood pressure measurement and, when available, anthropometric inputs for BMI calculation. Structured user inputs include brief demographic characteristics and selected NCD risk factor questions. The AI component applies rule-based stratification or decision logic to generate individualized, nondiagnostic outputs, including color-coded health indicators (eg, within-range vs elevated values), brief lifestyle guidance, and prompts encouraging users to seek formal screening or consultation when values fall outside recommended thresholds. Where enabled by the platform, a summary report could be generated and shared electronically with participants. The system is not intended for diagnosis or clinical decision-making. As the DigiHealth kiosk has not yet undergone independent clinical validation, all device-generated values in this study were treated strictly as informational prompts to elicit user perceptions and responses. Outputs were not interpreted as accurate clinical measurements, and no clinical decisions were made based on kiosk results. Participants were explicitly informed, both on-screen and verbally, that all outputs were informational only and required confirmation by licensed health care providers before any action.

### Implementation Phases

The kiosk deployment occurred in 2 operational phases as part of routine implementation logistics. During phase 1, two kiosks were deployed simultaneously and functioned as intended throughout the scheduled wellness activities. During phase 2, only 1 kiosk was used operationally. The second unit was temporarily withdrawn from use after an isolated instance of atypical output prompted recalibration procedures. As a precautionary measure, data collection continued using a single kiosk to ensure consistency and uninterrupted implementation activities. Operational differences between phases primarily affected workflow logistics, including queuing time and throughput, particularly during peak participation periods. These differences were managed on-site through scheduling adjustments and staff assistance to maintain an orderly participant flow.

### Data Collection

Two sources of data were collected:

1. Postuse survey data, assessing usability, perceived reliability, trust, privacy concerns, and immediate self-reported actions following kiosk use
2. Optional open-ended responses, allowing participants to provide brief comments on their experience

No clinical diagnoses, treatment decisions, or medical interventions occurred during the study. Participants who received kiosk outputs categorized as “outside recommended thresholds” were advised, through standardized on-screen messages and verbal guidance from trained staff, to seek a confirmatory evaluation from a licensed health care provider. Referrals followed existing institutional pathways and were presented as routine components of standard care.

### Analysis

The analyses were mainly descriptive and exploratory. Categorical variables were summarized using frequencies and percentages. Open-ended responses were reviewed to provide examples of user feedback. No inferential analyses were conducted to evaluate diagnostic accuracy, clinical outcomes, or causal relationships. The study was neither designed nor powered to compare acceptability outcomes between different implementation phases. Acceptability data were analyzed descriptively across the entire sample to reflect the overall user experience with the kiosk system. Although operational differences between phase 1 and phase 2 affected workflow efficiency, no formal comparison of acceptability metrics, such as satisfaction, perceived reliability, or privacy concerns, was conducted for each phase. This is a limitation of this study and points to an area for future research, especially regarding scalability and resource efficiency.

### Ethical Considerations

The Zamboanga City Medical Center Ethics Review Board approved this study under the assigned case protocol codes ZCMC-ERB-2025-34 and SPN-2025-06. The ethics review board approved the study solely as an implementation and acceptability assessment of an informational, nondiagnostic device. Participants were informed that the results required confirmation and were provided with standard referral guidance. Participants provided informed consent, with clear notices that kiosk outputs were informational, not diagnostic. Abnormal results were flagged on-screen, and medically trained staff provided verbal guidance and referral recommendations. Participants with abnormal results were advised to seek follow-up evaluation at a hospital. Trained staff explained the kiosk’s purpose and limitations and addressed questions to reduce anxiety and misinterpretation. Follow-up actions were voluntary. Participation was voluntary, and no financial compensation or incentives were provided. All collected data were anonymized, securely stored, and managed in accordance with the Philippine Data Privacy Act of 2012 (Republic Act 10173) [1].

## Results

### Participant Characteristics

A total of 384 public school teachers participated in the study. Most participants were female (n=312, 81.3%), whereas 18.8% (n=72) were male. The median age was 44 (IQR 37‐52) years, and the median length of service was 17 (IQR 10‐24) years. Table 1 summarizes the participants’ demographic characteristics.

**Table 1.** Sociodemographic profile of participants (N=384).

Variable	Participants, n (%)
Sex	
Female	312 (81.2)
Male	72 (18.8)
Age group (y)	
25‐34	48 (12.5)
35‐44	102 (26.6)
45‐54	95 (24.7)
55‐64	83 (21.6)
≥65	56 (14.6)
Length of service (y)	
<10	84 (21.9)
10‐19	124 (32.3)
20‐29	92 (24)
≥30	84 (21.9)

### Implementation of the AI-Enabled Health Kiosk

The AI-enabled health kiosk was implemented in 2 phases during workplace wellness activities at public schools from July to October. Phase 1 (July to August) involved deploying 2 kiosks, enabling parallel use, and reducing wait times during peak periods. Phase 2 (September to October) used a single kiosk, which increased queues and wait times during periods of high demand. Deployment was coordinated with school administrators and integrated into routines to minimize disruption. Kiosks were installed in accessible areas, and participants used them individually according to on-screen instructions. Throughout both phases, physicians and trained staff were present to explain the nondiagnostic nature of outputs, answer questions, and reinforce advisories about confirmatory evaluations for flagged outputs. Operational challenges included internet issues; variable wait times, especially during phase 2, when only 1 machine was functioning; and crowd management in a limited space. Assistance was required for some users, and privacy was addressed by minimizing screen visibility and providing verbal reminders, although some concerns persisted. The deployment demonstrated that the kiosk could be integrated into wellness activities under resource constraints, highlighting logistical challenges related to device availability and support in nonclinical settings.

### User Acceptability and Experience

User acceptability was high, with 85.7% (162/189) of the participants rating it as “good” or “excellent.” Only 9.5% (18/189) rated it as “fair,” and 4.8% (9/189) rated it as “poor” or “very poor.” Usability was rated very highly, with 93.1% (176/189) of the participants finding it “simple and easy to use” and the instructions clear, with a median score of 5 (IQR 4-5). Perceived reliability was also high, with a median score of 5; 86.8% (164/189) of the participants trusted the results, although 13.2% (25/189) were neutral or doubtful due to discrepancies. Privacy concerns were significant, with 31.7% (60/189) of the participants seeking stronger safeguards, such as partitioned spaces and clearer protocols.

### Illustrative Feedback on User Experience

Optional open-ended responses highlighted perceived convenience, accessibility, and time efficiency of the kiosk. Some participants noted operational challenges, including queuing, intermittent connectivity, and the need for on-site assistance. These comments are presented illustratively and were not subjected to formal qualitative analysis.

### Immediate Self-Reported Actions

After 1 to 2 weeks of kiosk use, 70.9% (134/189) of the participants reported consulting a health care professional, and 66.7% (126/189) reported making lifestyle changes, such as dietary adjustments or increased physical activity. Additionally, 82.5% (156 /189) of the participants shared their results with family members or coworkers. A small portion of the participants (33/189, 17.5%) reported no follow-up action, mainly due to uncertainty about the next steps or a perceived lack of urgency.

## Discussion

### Principal Findings

This cross-sectional implementation study showed that an AI-enabled health kiosk can be introduced in a workplace setting within a low-resource urban environment and is generally well received by users. High usability scores and positive user perceptions align with previous evidence indicating that integrated health kiosks can promote health engagement when incorporated into routine, nonclinical environments such as workplaces and community settings [2-4]. The presence of immediate self-reported actions after kiosk use, such as consulting health care providers and making lifestyle changes, suggests short-term behavioral activation. Similar trends have been observed in other workplace and community-based digital health and kiosk deployments, where exposure to personalized health information prompts reflection and initial action rather than sustained change [5-8]. Importantly, in line with methodological guidance on cross-sectional studies, these findings should not be interpreted as evidence of causal or long-term behavioral effects [9-12].

### Interpretation

The findings should be interpreted in the context of a nonclinical, informational intervention. The kiosk outputs used in this study were not clinically validated and were not intended to serve as diagnostic tools. As a result, the user responses observed are best seen as reactions to increased health awareness rather than responses to verified clinical risk. This distinction is well recognized in the literature on digital screening tools, self-testing technologies, and early-stage AI health systems, where informational outputs may encourage engagement without serving as diagnostic evidence [13-15].

### Privacy and Trust in Workplace-Based Digital Health

Privacy concerns raised by approximately one-third of the participants (63/189, 33.3%) align with earlier research on multiuser health kiosks and digital health systems, especially in work or semipublic environments, where perceived surveillance, employer oversight, and potential data misuse can undermine trust [4,16-18]. In workplace settings, worries are not only about data security but also about how health information might be accessed, interpreted, or used to influence employment-related decisions. These findings underscore the importance of clearly distinguishing health awareness tools from formal occupational health surveillance systems. In addition to transparency, several practical, workplace-specific measures can help reduce these concerns. These include explicit preuse statements clarifying that individual-level data are not accessible to school administrators or employers; visible on-site signage explaining data handling, storage duration, and deletion policies in plain language; and the use of anonymized or aggregated reporting formats when institutional summaries are created. Allowing participants to directly control whether a summary report is generated or shared, such as through an opt-in format rather than default sharing, may further enhance perceptions of autonomy and trust. Overall, these measures address not only technical privacy but also perceived boundaries between personal health information and employer oversight that are essential in employment-based digital health initiatives [19,20].

### Contextualization Within AI-Enabled Health Technologies

The DigiHealth kiosk falls into a broader category of task-focused, assistive AI tools created to aid human decision-making instead of replacing clinical judgment. Similar design principles are seen across various AI-powered health technologies, including computer vision and deep learning systems used in digital imaging and orthodontics, such as Dynasmile and Smile.AI. These systems focus on usability, engagement, and workflow integration during early deployment phases, often before formal clinical validation [13,21,22]. Across AI health domains, early-stage tools usually follow a phased development process, starting with assessments of implementation feasibility and acceptability before moving on to validation, effectiveness, and outcome evaluation [23-25]. Importantly, this study is within this early implementation phase and does not suggest that all AI systems are clinically ready, high-performing, or legally approved at this stage. Instead, it provides evidence on how users perceive and interact with assistive AI technologies when they are introduced into everyday workplace settings.

### Implementation Implications

From an implementation perspective, this study highlights several practical considerations for deploying AI-enabled health kiosks in workplace settings. First, usability and interface simplicity remain essential for adoption, particularly in environments with varying levels of digital literacy. Previous kiosk research consistently demonstrates that clear visual cues, minimal text, and intuitive navigation enhance engagement and reduce user uncertainty [4,16,26].

Second, having physicians or medically trained staff on-site likely increased trust and helped ensure proper understanding of kiosk outputs. Human assistance acts as an important safeguard in digital health implementations by providing context to automated information, answering questions in real time, and emphasizing that the outputs are not diagnostic [13,24,27,28].

Third, privacy governance in workplace deployments needs more than just technical safeguards. Clearly separating health awareness initiatives from employment evaluation processes through explicit institutional policies should be both communicated and enforced visibly. Practical steps might include written assurances supported by both implementers and host organizations, clear role definitions that prevent supervisors from accessing individual data, and standardized communication scripts used by staff to consistently explain these boundaries. These measures are especially important in public sector workplaces, where hierarchical structures can heighten concerns about surveillance or coercion.

Finally, the findings of this study are specific to a single occupational group in an urban Philippine setting and involved phased deployment with varying device availability. Therefore, the results cannot be generalized to other populations, rural areas, or health systems without further research. Success in implementation largely depends on institutional culture, workforce dynamics, and access to follow-up care. Future studies should investigate how privacy perceptions and acceptability differ across various employment settings and explore governance models that balance scalability with trust and ethical deployment [29,30].

### Limitations

This study has several important limitations that should be considered when interpreting the results. First, the AI-enabled health kiosk used in this study has not been independently validated against gold standard laboratory or clinical reference methods. Therefore, device-generated outputs should not be regarded as accurate biomedical measurements or used to estimate disease prevalence, metabolic risk, or diagnostic accuracy. The study was mainly designed to assess implementation and acceptability, rather than clinical performance, so the conclusions are limited [13,15,24]. Second, the cross-sectional design restricts findings to a single point in time and does not allow evaluation of sustained behavior change, adherence, or long-term clinical outcomes. Actions taken after kiosk use were self-reported and immediate; thus, causal relationships cannot be established [9-13,31]. Self-reported data are also prone to recall bias and social desirability bias, which may influence estimates of postuse actions [12]. Third, the study was conducted among a single occupational group, public school teachers, in an urban area of the Philippines, using a phased rollout with limited device availability. These contextual factors limit the extent to which the findings can be generalized to other populations, rural areas, informal workers, or health systems with different organizational or cultural features [32]. Fourth, although helpful user feedback was collected through optional open-ended survey responses, the study did not use a formal qualitative methodology. Therefore, the qualitative findings serve to provide context for the quantitative data rather than detailed thematic analysis. Finally, although physicians and medically trained staff were present during implementation and abnormal outputs were referred through standard hospital care pathways, the study did not track clinical follow-up outcomes.

### Implications for Practice and Future Directions

Despite these limitations, the study provides important insights for designing and evaluating AI-powered health kiosks in workplaces and nonclinical settings. From an implementation perspective, the results emphasize the importance of usability, on-site support, and clear communication about data privacy and limitations. Having medically trained staff appears to assist in accurately interpreting informational outputs and may be vital in early deployments [14,24,27]. Privacy concerns voiced by many participants highlight the need for trust-building strategies that go beyond compliance with data regulations. Specific data encryption, storage, and anonymization protocols should be strictly enforced to ensure adherence to Republic Act 10173. Clear explanations of data flows, visible safeguards, and a distinct separation between health data and employer oversight are crucial for successful adoption in workplaces [17-20]. Future research should focus on formal clinical validation of these kiosks, including assessments of accuracy, precision, sensitivity, and specificity, as well as comparisons with gold standard laboratory and clinical methods. Such validation is essential before evaluating diagnostic usefulness, public health impact, or the integration of these kiosks into clinical or screening programs [15,23]. Long-term and mixed methods studies are also vital for understanding ongoing engagement, behavioral maintenance, and health outcomes after use. Using validated behavioral models, standardized acceptability measures, and follow-up evaluations can enhance causal and theoretical understanding [7,31,33]. Finally, comparative implementation research across occupational groups, regions, and resource levels can help identify factors that influence the successful deployment and scaling of AI-based health kiosks across various settings [4,16].

### Conclusions

This cross-sectional implementation study shows that an AI-enabled health kiosk can be feasibly deployed and is generally well accepted in a workplace setting within an urban, low-resource public school context. High usability scores and positive user perceptions suggest that such kiosks may support health awareness and short-term engagement when incorporated into routine workplace wellness activities. However, these results are preliminary and specific to this context. The study did not evaluate diagnostic accuracy, disease prevalence, or clinical effectiveness, so it cannot draw conclusions about causal or long-term behavioral or health outcomes. The immediate self-reported actions following kiosk use probably reflect increased awareness rather than responses to validated clinical risk. Notably, the presence of privacy concerns among many participants highlights the importance of careful design and governance when deploying digital health tools in employment settings. Future implementations might benefit from concrete safeguards that directly address employer oversight concerns, such as anonymizing data at collection, avoiding storage of identifiable individual data, and providing clear on-screen assurances that results are not accessible to school administrators or employers. Additional steps, including optional user-controlled report generation (eg, personal printouts or direct delivery to individuals without institutional access), may further build trust and reinforce perceived autonomy. Overall, AI-enabled health kiosks can serve as informational and engagement tools in nonclinical workplace settings. However, their responsible use depends on thorough clinical validation, long-term assessment, and clear privacy protections that are openly communicated to users. Addressing ethical, trust, and governance issues, especially those related to separating health information from employment oversight, will be crucial before these technologies are widely deployed, used for population health, or integrated into official health systems.

## Abbreviations

AI: artificial intelligence
LMIC: low- and middle-income country
NCD: noncommunicable disease
